# Update on the Global Charter for the Public’s Health

**DOI:** 10.2471/BLT.17.198820

**Published:** 2018-05-14

**Authors:** Bettina Borisch, Marta Lomazzi, Michael Moore, Ruediger Krech

**Affiliations:** a World Federation of Public Health Associations & Institute of Global Health, University of Geneva, Campus Biotech, Chemin des Mines 9, 1202 Geneva, Switzerland.; b World Federation of Public Health Associations, Geneva, Switzerland.; c Health Systems and Innovation, World Health Organization, Geneva, Switzerland.

Rudolf Virchow described the links between the social, political and economic factors and health in 1848, when he reported on an outbreak of typhus fever in Upper Silesia.^1^ He was convinced that disease is socially and economically determined and that it should therefore be addressed at the social and economic levels. The social determinants of health he described in his report remain the main causes of ill-health today, whether from communicable or noncommunicable diseases.^2^

Against this background, the rapid global increase of inequities^3^ is distressing. Even in countries with a solid social system, there has been a strong increase in income polarization.^4^ The gap between rich and poor is growing exponentially in nearly every region of the world. In parallel, we observe important changes in the labour markets, which now go beyond national borders and classical jobs. Globalization has made political processes more complex and disaggregated, and makes good governance challenging.

The importance of strong governmental institutions had been underestimated because the focus was on economy. However, institutions systems and the rule of law are needed to manoeuvre the changes ahead.

The Global Charter for the Public’s Health^5^ is a joint effort of the World Federation of Public Health Associations, the World Health Organization (WHO) and multiple stakeholders to provide a comprehensive, clear and flexible framework to adapt public health to its global context. The charter provides a set of tools including services and enabler functions that help public health professionals and organizations to develop policies, take action and promote the conditions for healthy lives. These tools rely on a strategic approach that considers the political and economic context and priorities of each country.^6^^,^^7^ The charter focuses on the functions of information, capacity, advocacy and governance to promote the public’s health, increase health security and fulfil the human right to health (Fig. 1). Effective leadership in public health is important for applying the charter’s framework and achieving resilient, secure and accountable health systems.

**Fig. 1:**
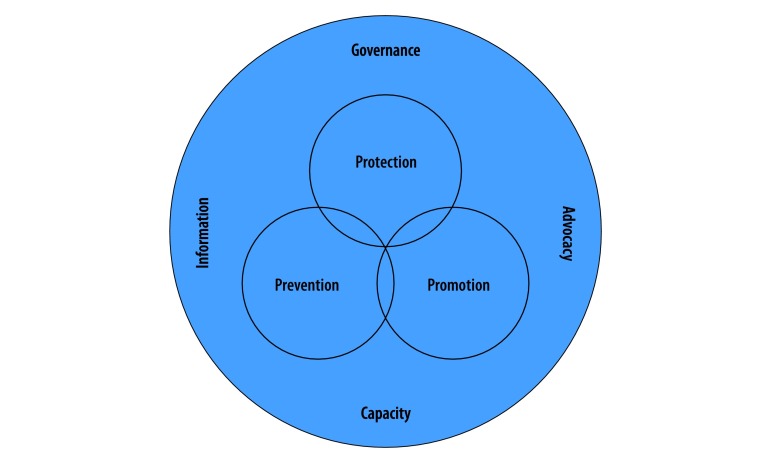
The global charter for the public’s health: core services

Note: Core services are protection, prevention and promotion. Enabler functions are information, capacity, advocacy and governance.

Data source: Republished from World Federation of Public Health Associations.^5^

Various organizations, in different settings, have already implemented the charter’s framework. The Commonwealth of Nations has developed toolkits for the implementation of frameworks based on the charter, such as, A systems framework for healthy policy^8^ and Health protection policy.^9^ WHO Regional Office for Europe is using the charter’s framework as part of its strategy on public health functions. Several members of the World Federation of Public Health Associations have adopted the charter in a range of ways. In Australia, the charter has been used to reshape the strategic plan of the national public health associations and has been incorporated into the teaching schools of public health and in the work of State and Federal governments.^10^ Panama has integrated the charter in Generation Healthy Panama 2030^11^ and Mongolia is developing its health charter based on the charter’s framework. The charter was cited in the Vienna Declaration, the main output of the European Public Health Conference in 2016, as a tool to renew and expand global commitment to health. The World Federation of Public Health Associations and Cameroon and South Africa’s Public Health Association have focused their public health congresses on the charter. Maastricht University has included the charter in one of its master’s programmes and other public health schools are now doing the same.^12^

The charter’s function of information should entail innovative surveillance and monitoring approaches. These approaches require mechanisms to assess the social, economic and commercial determinants that influence the health of different population groups, whether at the country or global level.^13^ Effective monitoring and evaluation relies on well-functioning country health information systems that are built on accountable, accurate and evidence-based resources, such as civil registration and vital statistics systems, health facilities, administrative sources, surveillance systems and surveys.^14^ However, health information systems in many countries do not have, or do not use, reliable information. Implementing the United Nations’ *Transforming our world: the 2030 agenda for sustainable development*^15^ will require innovative and reliable data acquisition and integration approaches to improve the availability, quality, timeliness and disaggregation of data. Appropriate dissemination of reliable data will also increase awareness and knowledge and empower people and communities, which is the basis of public health actions.

Better governance for health can be achieved through appropriate public health legislation. We need instruments that enable joined-up government, that is, whose departments communicate effectively and coordinate policies, in a manner that helps to address the interconnectedness of health with decisions in non-health sectors. For example, the Melbourne Demand for Action^16^ is consistent with the charter and calls on public health associations and governments to recognize the impact of legislation on health. The Melbourne Demand calls for the implementation of legislation, regulation and taxation on unhealthy commodities and opposition to international treaties that exacerbate health inequities and poverty, or are inconsistent with poverty reduction. This demand for action also calls for opposing civil and other wars that displace large groups of people and exclusionary processes that disadvantage entire regions, countries, populations or communities.

Recent global health emergencies provided ample evidence that the public health systems to protect the public from health hazards need improvement. Preparedness efforts need to be better integrated in national health systems to assist countries to comply with the WHO International Health Regulations (2005), for instance through Joint Evaluation Exercises.

Efforts for the prevention of disease and health promotion are core functions of public health. These functions should contribute to identifying and addressing major risk factors and their underlying causes, and to addressing the health inequities within and between countries.

Ethical advocacy is a fundamental function of public health. Effective advocacy is based on the values of solidarity, universalism and equity. This entails engaging with the community and using social mobilization mechanisms and communication.

Achieving these health objectives will require appropriate capacity. Some public health capacities are well established, but others are still rarely found in public health training curricula. To improve capacity building, basic public health teaching with a special focus on the SDGs should be integrated in all curricula and in awareness raising for policy-makers. Building capacity also requires workforce planning, decent work conditions and salaries, as well as infrastructures.

The implementation of the charter’s functions with a flexible approach could contribute to achieving the SDGs, with a potential impact in reducing inequities. However, the health sector will need leadership, commitment and ownership at all levels to successfully implement the charter.

## References

[1] Virchow RC (Rather LJ, editor). Collected essays on public health and epidemiology. Volume 1 Boston: Science History Publications; 1985 pp. 204–319.

[2] Marmot M. Global action on social determinants of health. Bull World Health Organ. 2011 10 1;89(10):702. 10.2471/BLT.11.094862 22084501 PMC3209984

[3] Alvaredo F, Chancel L, Piketty T, Saez E, Zucman G. World inequality report 2018. Paris: World Inequality Lab; 2018. Available from: http://wir2018.wid.world/files/download/wir2018-summary-english.pdf [cited 2018 Apr 11].

[4] Grabka M, Goebel J, Schröder C, Schupp J. Shrinking Share of Middle-Income Group in Germany and the US. DIW Econ Bull. 2016 5 6;18:199–211.

[5] Global Charter for the Public’s Health [internet]. Geneva: World Federation of Public Health Associations; 2016. Available from: https://www.wfpha.org/wfpha-projects/14-projects/171-a-global-charter-for-the-public-s-health-3 [cited 2018 Apr 9].

[6] Moore M, McKee M, Borisch B, Ricciardi W. The Global Charter for the Public’s Health. Eur J Public Health. 2016 4;26(2):207. 10.1093/eurpub/ckw013 27009898

[7] Lomazzi M. A Global Charter for the Public’s Health-the public health system: role, functions, competencies and education requirements. Eur J Public Health. 2016 4;26(2):210–2. 10.1093/eurpub/ckw011 26956023 PMC4804738

[8] A systems framework for healthy policy. Advancing global health security and sustainable well-being for all. London: Commonwealth Secretariat; 2016. Available from: https://drive.google.com/file/d/0B8wr6920su0aeXNVR01IeHdTYmc/view [cited 2018 Apr 11].

[9] Health protection policy kit: health as an essential component of global security, second edition. London: Commonwealth Secretariat; 2017. Available from: https://www.thecommonwealth-healthhub.net/wp-content/uploads/2017/05/HPToolkitwordversionEd2-CHMM-2017.pdf [cited 2018 Apr 11].

[10] Public Health Association of Australia strategic plan. Curtin: Public Health Association of Australia Inc; 2017. Available from: https://www.phaa.net.au/documents/item/2042 [cited 2018 March 6].

[11] Melo GE. Presentan propuesta país “Generación Panamá Saludable 2030” [internet]. Oslo: UX Themes; 2018. Spanish. Available from: https://saludpublicapanama.org/2018/02/28/presentan-propuesta-pais-generacion-panama-saludable-2030/ [cited 2018 Apr 11].

[12] Charter implementation. Geneva: World Federation of Public Health Associations; 2017. Available from: http://www.wfpha.org/charter/charter-implementation#public-health-associations-implementation [Cited 2017 Sept 28].

[13] Welcome to the sustainable development goal indicators website. New York: United Nations; 2018. Available from: https://unstats.un.org/sdgs/ [Cited 2018 March 6].

[14] Country data, universal accountability. Monitoring priorities for the global strategy for women's, children's and adolescents' health (2016–2030). Geneva: World Health Organization; 2016. Available from: http://www.who.int/life-course/partners/global-strategy/gs-monitoring-readiness-report.pdf [cited 2018 March 6].

[15] Resolution A/RES/70/1. Transforming our world: the 2030 agenda for sustainable development. In: Seventieth United Nations General Assembly, New York, 25 September 2015. New York: United Nations; 2015. Available from: http://www.un.org/ga/search/view_doc.asp?symbol=A/RES/70/1&Lang=E [cited 2017 Nov 3].

[16] World Federation of Public Health Associations demand for action – Melbourne 2017. Geneva: World Federation of Public Health Associations; 2017. Available from: http://www.wcph2017.com/d/WCPH2017-Melbourne-Demand-for-Action.pdf [cited 2018 March 7].

